# Prevalence and Predictive Factors for Upfront Dose Reduction of the First Cycle of First-Line Chemotherapy in Older Adults with Metastatic Solid Cancer: Korean Cancer Study Group (KCSG) Multicenter Study

**DOI:** 10.3390/cancers13020331

**Published:** 2021-01-18

**Authors:** In Gyu Hwang, Minsuk Kwon, Jin Won Kim, Se Hyun Kim, Yun-Gyoo Lee, Jin Young Kim, Su-Jin Koh, Yoon Ho Ko, Seong Hoon Shin, Soojung Hong, Tae-Yong Kim, Sun Young Kim, Hyun Jung Kim, Hyo Jung Kim, Myung Ah Lee, Jung Hye Kwon, Yong Sang Hong, Kyung Hee Lee, Sung Hwa Bae, Dong-Hoe Koo, Jee Hyun Kim, In Sook Woo

**Affiliations:** 1 Department of Internal Medicine, Chung-Ang University Hospital, Chung-Ang University College of Medicine, Seoul 06973, Korea; oncology@cau.ac.kr (I.G.H.); means@naver.com (M.K.); 2 Department of Internal Medicine, Seoul National University Bundang Hospital, Seoul National University College of Medicine, Seongnam 13620, Korea; whunt@daum.net (J.W.K.); ksh78md@naver.com (S.H.K.); jhkimmd@snu.ac.kr (J.H.K.); 3 Department of Internal Medicine, Kangbuk Samsung Hospital, Sungkyunkwan University School of Medicine, Seoul 03181, Korea; gosciny@gmail.com (Y.-G.L.); dhkoo.smc@gmail.com (D.-H.K.); 4 Department of Internal Medicine, Keimyung University Dongsan Medical Center, College of Medicine, Keimyung University, Daegu 42601, Korea; takgu96@gmail.com; 5 Department of Hematology and Oncology, Ulsan University Hospital, University of Ulsan College of Medicine, Ulsan 44033, Korea; sujinkoh@hanmail.net; 6 Department of Internal Medicine, Eunpyeong St. Mary’s Hospital, College of Medicine, The Catholic University of Korea, Seoul 03476, Korea; koyoonho@catholic.ac.kr; 7 Department of Internal Medicine, Kosin University Gospel Hospital, Kosin University College of Medicine, Pusan 49267, Korea; ssh1533@hanmail.net; 8 Department of Internal Medicine, National Health Insurance Service Ilsan Hospital, Goyang 10444, Korea; suzzy901@nhimc.or.kr; 9 Department of Internal Medicine, Seoul National University Hospital, Seoul National University College of Medicine, Seoul 03080, Korea; ktyongmd@gmail.com; 10 Department of Oncology, Asan Medical Center, University of Ulsan College of Medicine, Seoul 05505, Korea; cathykimmd@gmail.com (S.Y.K.); yshong@amc.seoul.kr (Y.S.H.); 11 Department of Internal Medicine, Soonchunhyang University Bucheon Hospital, Soonchunhyang University College of Medicine, Bucheon 14584, Korea; khjbless@hanmail.net; 12 Department of Internal Medicine, Hallym University Sacred Heart Hospital, Hallym University College of Medicine, Anyang 14068, Korea; hemonc@hallym.or.kr; 13 Department of Internal Medicine, Seoul St. Mary’s Hospital, Cancer Research Institute, The Catholic University of Korea, Seoul 06591, Korea; angelamd@catholic.ac.kr; 14 Department of Internal Medicine, Chungnam National University College of Medicine, Daejeon 35015, Korea; kwonjhye.onco@gmail.com; 15 Department of Internal Medicine, Yeungnam University Medical Center, Yeungnam University College of Medicine, Daegu 42415, Korea; lkhee@med.yu.ac.kr; 16 Department of Internal Medicine, Daegu Catholic University Hospital, Daegu Catholic University College of Medicine, Daegu 42472, Korea; sunghwa@cu.ac.kr; 17 Division of Medical Oncology, Department of Internal Medicine, Yeouido St. Mary’s Hospital, College of Medicine, The Catholic University of Korea, Seoul 07345, Korea

**Keywords:** predictive, dosing, chemotherapy, older adults

## Abstract

**Simple Summary:**

Arbitrary upfront dose reduction (UDR) of palliative chemotherapy has often been performed according to the judgement of the physician of older adults with metastatic solid cancer in current practice. UDR might decrease treatment efficacy in older adults but may be helpful for palliation, so selecting older adults who benefit from UDR and the identification of predictors of UDR are required. The authors investigated the prevalence and predictors of UDR through variables of geriatric assessment (GA). Chemotherapy compliance between the UDR and standard dose patient groups was also compared. The results of this study demonstrated that approximately 60% of older adults with metastatic solid cancer received UDR. Poor performance status (PS) and living without a spouse were predictive factors of UDR of first-line palliative chemotherapy, and patients with UDR better-tolerated chemotherapy compared with patients with standard doses.

**Abstract:**

Old age alone does not reflect an intolerability to chemotherapy. However, upfront dose reduction (UDR) of the first cycle of first-line palliative chemotherapy has sometimes been chosen by physicians for older adults with metastatic cancer due to concerns regarding adverse events. The development of predictive factors for UDR of palliative chemotherapy would be helpful for treatment planning among older adults. This was a secondary analysis of a study on predicting adverse events of first-line palliative chemotherapy in 296 patients (≥70 years) with solid cancer. We assessed the prevalence of UDR of the first cycle of first-line chemotherapy and the association of UDR with the variables of geriatric assessment (GA) and chemotherapy compliance. Among the 296 patients, 177 (59.8%) patients were treated with UDR. The mean percentage of UDR for the total patient group was 19.2% (range: 4–47%) of the standard dose. In a multivariate analysis, poor performance status (PS) and living without a spouse were independent predictive factors of UDR of first-line palliative chemotherapy in older adults. Patients with UDR showed fewer grade 3–5 adverse events versus the standard dose group. Study completion as planned was significantly higher in the UDR group versus the standard dose group. Older adults with UDR better tolerated chemotherapy than patients with a standard dose.

## 1. Introduction

Finding the balance between the potential benefits and harm from anticancer treatments is a major issue in the care and management for frail and older adults with cancer. The optimal dose and schedule of chemotherapy for older cancer patients have not been established so far due to the underrepresentation of older adults in clinical trials, although about half of cancer patients are over 70 years old in the United States [1,2,3]. Older adults aged 65–74 years account for less than 25% of enrolled patients in therapeutic clinical trials, while patients age 75 years and older represent less than 10% of patients [2].

The modification of doses and schedules for chemotherapy is one therapeutic strategy for older adults with cancer [4]. In clinical practice, arbitrary dose reduction during the first cycle of chemotherapy can be applied to decrease the side effects of systemic chemotherapy because of concerns that older adults might not tolerate the drugs. Few data, however, are available on how many older adults are treated with upfront dose reduction (UDR) at the first cycle of first-line palliative chemotherapy. Furthermore, there is currently a lack of rationale or guidelines on UDR at the first cycle of palliative chemotherapy in older adults with cancer [4,5]. Whether the UDR of chemotherapy decreases toxicities and improves the quality of life in older adults with advanced cancer remains unclear.

Contrary to popular concerns in clinical practice, some older adults can tolerate chemotherapy well, and the UDR of chemotherapy for these patients can compromise their efficacy. In 2015, Gajra et al. reported the prevalence of dose reduction of chemotherapy during the first cycle of chemotherapy of multiple lines and the clinical factors associated with dose reduction in patients 65 years and older with solid tumors. The authors found that chemotherapy dose reduction during the first cycle was performed in 15% of patients receiving adjuvant chemotherapy and 25% receiving palliative chemotherapy [5].

Although numeric age alone does not reflect a tolerability to cytotoxic chemotherapy, older adults with cancer are generally considered to be more vulnerable to the adverse effects of systemic chemotherapy than younger patients. This may be because older adults may have a decline in function of their major organs, such as the kidneys, liver, and bone marrow and physiological changes of decreased muscle mass and more chances of comorbid illness [6]. When planning the schedules and doses of systemic chemotherapy before the administration of chemotherapeutics, physicians must consider the goal of treatment (curative intent, such as neoadjuvant or adjuvant chemotherapy or palliation), drug sensitivity, tumor burden and biology, characteristics and toxicities of chemotherapeutic agents, performance status, and concomitant disease, in addition to the age of the patient. Although tools to identify risk factors for severe toxicity with chemotherapy for older adults with cancer have been developed, the optimal dosing and scheduling of chemotherapy for older adults with cancer have not been developed [7,8,9]. Gajra et al. reported that comorbid illnesses such as renal or hepatic dysfunctions were associated with dose reduction during the first cycle in patients with palliative chemotherapy [5]. The study included more frail patients who received multiple lines of chemotherapy.

Quality of Life (QOL) and disease control rates can be other important issues in older adults with metastatic cancer, and UDR could be beneficial for older adults in a palliative setting. However, the arbitrary UDR of systemic chemotherapy may decrease the dose intensity and compromise the efficacy or the overall response rate of chemotherapy, and thus, it is not recommended in an adjuvant setting. To the best of our knowledge, the association between UDR of the first cycle of first-line palliative chemotherapy and the clinical parameters through a geriatric assessment (GA) for older Asian adults newly diagnosed with cancer has not been examined.

This study is a secondary analysis of a prospective observational study that validated a geriatric screening tool and estimated toxicities through a GA and the clinical factors associated with first-line systemic chemotherapy for older adults with metastatic solid cancers [9,10]. We evaluated the prevalence of UDR among the total older adult group ≥ 70 years of age. We then examined the association of the clinical variables, including age; tumor type; performance status; stage; the variables of the GA (Charlson Comorbidity Index, activities of daily living (ADL), instrumental ADL, cognitive function, depression, delirium risk screening, nutritional status, and living alone or with spouse); and adverse effects, with UDR.

## 2. Results

### 2.1. Patients’ Characteristics

The baseline characteristics of the total 296 patients are summarized in Table 1. The median age of all patients was 75 years (range 70–93 years), and 41 patients (13.9%) were older than 80 years. There were 205 (69%) male patients, and 81% of patients had a good performance status with the Eastern Cooperative Oncology Group (ECOG) ≤ 1. UDR was done for 177 patients (59.8%). The mean percentage of the UDR for the total patient group was 19.2% (range: 4–47%). The patients with a poor performance status (ECOG PS 2~4, *p* < 0.01) were more likely to receive chemotherapy with an UDR. Patients who received an UDR were more likely to be older, although a statistical significance was not observed. There were no significant differences in the other clinicopathological parameters, such as gender, comorbid illnesses such as hypertension and diabetes mellitus, or cancer type, according to the UDR.

Colorectal cancer was diagnosed in 84 patients (28.4%), lung cancer in 74 patients (25.0%), biliary cancer in 33 patients (11.1%), and gastric cancer in 32 patients (10.8%). Among the total patient group, 274 patients (91%) received combination chemotherapy, 24 patients (8%) received monotherapy, and 3 patients (1%) received an unknown treatment. Patients with colorectal cancer were more likely to receive UDR, whereas patients with lung cancer, biliary cancer, pancreatic cancer, or gastric cancer were more likely to receive standard dose chemotherapy.

We also assessed the laboratory markers that were performed before chemotherapy: hemoglobin, white blood cell (WBC), platelet, aminotransferase, albumin, the estimated glomerular filtration rate (eGFR), and c-reactive protein. These markers were not significantly different between the UDR and standard dose groups.

### 2.2. Association between UDR and the Variables of the GA

In comparison with patients treated with standard dose chemotherapy, patients who received chemotherapy with UDR were more dependent during activities of daily living (35.0% vs. 19.3%, *p* < 0.01), had a higher delirium risk (9.6% vs. 1.7%, *p* < 0.01), and had a higher likelihood of living without a spouse (33.3% vs. 19.3%, *p* < 0.01). In addition, male patients living without a spouse were more likely to receive chemotherapy with UDR (19.8% vs. 7.9%, *p* = 0.02). There were no significant differences in the other GA variables (Table 2). In the multivariable analysis, a poor ECOG PS (odds ratio (OR) 2.33; 95% confidence interval (CI) 1.17–4.64, *p* = 0.02) and living without a spouse (OR 1.89; 95% CI 1.07–3.33, *p* = 0.03) were independently associated with UDR (Table 3).

### 2.3. Changes in Adverse Events According to UDR

Grade 3–5 adverse events were observed more frequently in patients receiving standard doses than in patients with UDR (63.9% vs. 48.6%, *p* = 0.01). Patients who received a standard dose were more likely to develop hematologic adverse events (neutropenia ≥ grade 3, 37.0% vs. 20.9%, *p* < 0.001, anemia ≥ grade 3, 15.1% vs. 8.5%, *p* = 0.04, and thrombocytopenia ≥ grade 3, 11.8% vs. 5.1%, *p* = 0.04). Nonhematologic adverse events occurred at similar frequencies in both groups, except for generalized muscle weakness (5.9% vs. 0.6%, *p* = 0.01) and oral mucositis (0% vs. 4.0%, *p* = 0.04) (Table 4).

### 2.4. Effect of Cisplatin Containing Chemotherapy on Hematologic Adverse Events

When we compared the hematologic adverse events according to cisplatin-containing chemotherapy between the two groups, the standard dose group revealed a significantly higher frequency of grade 3–5 neutropenia, anemia, or thrombocytopenia compared to the UDR group. The frequency of cisplatin-containing chemotherapy regimens was substantially linked to the cancer type. In our cohort, 90.9% of biliary tract cancer patients (*N* = 30), 100% of head and neck cancer patients (*N* = 8), and 41.9% of lung cancer (NSCLC and SCLC) patients (*N* = 49) were receiving cisplatin. However, 98.8% of colon cancer patients (*N* = 83) were not receiving cisplatin (Table 5).

### 2.5. Tolerance and Compliance According to UDR

Patients receiving a standard dose had a higher incidence of emergency room (ER) visits or hospitalization (52.9% vs. 37.9%, *p* = 0.01). There was no significant difference between the UDR and standard dose groups with respect to the delayed number of cycles and delayed days of chemotherapy. However, patients with UDR were more likely to complete the expected cycles of chemotherapy than patients with a standard dose (29.4% vs. 16.8%, *p* = 0.02) (Table 6 and Table 7).

### 2.6. Overall Survival (OS) and Progression-Free Survival (PFS)

**Figure 1:**
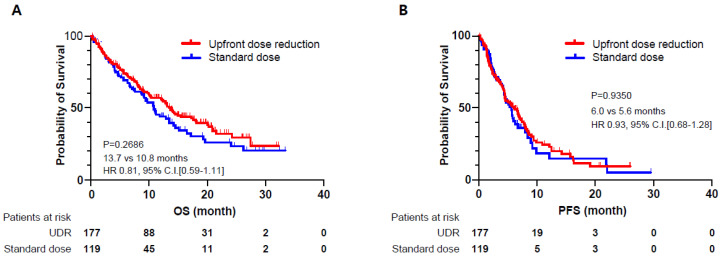
Kaplan-Meier curves for overall survival (OS) and progression free survival (PFS) between the standard dose group and the group with upfront dose reduction (UDR). (**A**) overall survival (**B**) progression free survival. CI, confidence interval; HR, hazard ratio.

The UDR group tended to demonstrate longer OS and PFS, but they were not statistically significant. The types of cancers and chemotherapeutic regimens of the same cancers were very heterogenous in this study (Figure 1).

### 2.7. Changes in the Relative Dose Intensity (RDI)

**Figure 2:**
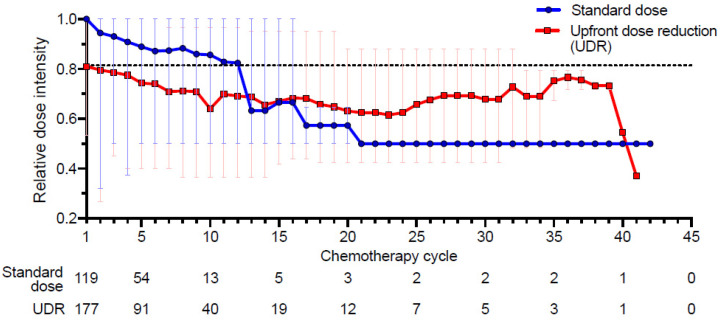
Changes in relative dose intensity (RDI) of the standard dose and UDR groups. The dotted line demonstrates each mean RDI chemotherapy cycle, and the error bar demonstrates RDI ranges.

The longitudinal changes in the RDI were compared between the standard dose chemotherapy and UDR groups. The mean RDI of the second cycle significantly decreased in the standard dose group compared to the UDR group (Figure 2).

## 3. Discussion

Human life expectancy is progressively increasing, and the population of older adults with cancer is growing worldwide [11,12]. Among older or frail patients with metastatic cancer, it may be challenging to identify and predict the patients who could benefit from anticancer therapeutic strategies without debilitating their QOL. Dosing and adjustments of the schedule for anticancer chemotherapy for appropriate candidates can be an important therapeutic strategy, because palliation to improve the QOL and minimizing toxicities following cytotoxic chemotherapy are goals of treatment for these patients.

Clinical trials on the optimal dose and schedule of chemotherapy for older or frail patients have been sparse so far, and older adults tend to be unfit or ineligible for the strict criteria of clinical trials [2,13]. When older adults meet the stringent criteria of clinical trials, they have recently been enrolled without a comprehensive GA in major clinical trials on first-line chemotherapy for metastatic cancer [14,15,16,17,18,19,20]. The unique biology, functional and nutritional status, comorbid illness, and psychologic states specific in older adults are underestimated in clinical trials.

Although chronological old age alone does not always reflect an intolerability to systemic cytotoxic chemotherapy in patients with cancer, in older adults, a functional decline of their major organs, including the intestines, liver, and kidneys, and comorbid illness of these major organs may cause a vulnerability to serious adverse reactions following systemic cytotoxic chemotherapy compared with younger patients [6]. Pharmacokinetic implications such as “a decline in renal function” should be distinguished from pharmacodynamic characteristics such as “a decline in bone marrow”. Indeed, UDR is particularly justified and is not associated with drug plasma underexposure in patients with a decline in renal function. However, in older adults with a decline in bone marrow, the drug’s therapeutic index is decreased, and the decision on a chemotherapy treatment may be questionable. Hemoglobin, WBC, platelets, and the estimated glomerular filtration rate (eGFR), which were performed before chemotherapy, were not significantly different between the UDR and standard dose groups in this study. The International Society of Geriatric Oncology (SIOG) suggested checking the creatinine clearance before starting chemotherapy and guide the dose adjustments in older adults with renal insufficiency [21]. Peterson et al. reported that decreased renal function and not serum creatinine alone was associated with chemotherapy toxicities regardless of the chemotherapy type in 500 patients aged 65 and older with cancer [22]. Renal function was evaluated using four methods in the study [23,24,25,26].

In terms of adjuvant chemotherapy for older adults, upfront dose adjustment is currently not recommended [5,27]. Arbitrary dose reduction may lead to negative outcomes for patients who receive adjuvant chemotherapy [4]. Healthy older adults receiving more aggressive adjuvant chemotherapy showed better disease-free survival and overall survival than those with less chemotherapy, such as younger patients [28]. Randomized controlled studies on the prediction of UDR during the first cycle of first-line chemotherapy for older adults with solid cancer are lacking, and few guidelines for the optimal dose reduction of the first cycle of first-line palliative chemotherapy for older adults are available at present. In clinical practice, a dose reduction depends on the discretion of the physician, who considers age, the comorbidities of major organs, the performance status, and the goal of chemotherapy.

In 2011, a randomized factorial trial for 459 patients with metastatic colorectal cancer who were unsuitable for standard dose chemotherapy was performed [29]. In these patients, 80% of the standard dose of chemotherapy was administered as the starting dose, and the dose was increased up to the standard dose after six weeks when the patients tolerated the chemotherapy. The patient ages ranged from 35 to 87 years old (median, 74 years), and frail patients less than 70 years old (*n* = 98) were also included in this study. A 117-item comprehensive health assessment, including physical parameters performed by a nurse and symptoms from the patients, was also obtained at the baseline and at 12 weeks for a response evaluation. The authors suggested that reduced the starting dose of chemotherapy led to a good patient-oriented outcome and palliative effects for frail or older adults [29].

In 2015, the prevalence of a primary dose reduction of chemotherapy (PDR) of the first cycle of chemotherapy and the relationship between the PDR and clinical factors were analyzed in 500 older patients (≥65 years) in the United States [5]. The patients received adjuvant (179 patients) or palliative chemotherapy (321 patients). This study was a secondary analysis of a multicenter study that evaluated a comprehensive GA predicting chemotherapy toxicity. First-line chemotherapy was administered for 190 out of the 321 patients (59.2%) who received palliative chemotherapy; the authors did not describe the details of the lines of palliative chemotherapy for the remaining 131 patients (40.8%). Dose reduction for the first-line or more palliative chemotherapy was done for 81 out of the 321 patients (25%) [5].

Age cutoffs for older adults with advanced cancer vary according to the study. In studies focused on patient-oriented outcome measures in palliative chemotherapy and functional declines during first-line chemotherapy of older adult with cancer, older adults were classified as 70 and older [30,31]. We investigated the association of the clinical parameters, including the GA variables and UDR of the first cycle of first-line palliative systemic chemotherapy, in older adults ≥70 years with solid tumors. Comparing patients who received only palliative chemotherapy in Gajra’s study with those in our study, there were considerable differences in the prevalence of dose reduction of the first cycle (25% vs. 59.8%), eligible age (≥65 years vs. ≥70 years), and the lines of chemotherapy (first-line or more vs. first-line only) between the two studies. In the study by Gajra et al., age, renal/hepatic disease, and other cancers were associated with a dose reduction of the first cycle of palliative chemotherapy [5]. However, we did not find a significant association of age and comorbid illness between the UDR and standard dose patient groups. We observed associations of the poor performance status, dependent activities of daily living (ADL), living without a spouse, and delirium risks among the variables of the GA with UDR during the univariate analysis, but poor ECOG PS and living without a partner were significantly associated with UDR during the multivariate analysis. The results showed that poor performance status and living without a spouse could become predictive factors for first-line palliative chemotherapy UDR in older adults.

The performance status is not objectively considered to be a predictor of tolerability for palliative cytotoxic chemotherapy, and the WHO performance status was not significantly associated with the overall treatment utility outcome during the multivariate analysis [29]. However, in our study, a poor ECOG performance status was associated with a dose reduction of the first cycle of first-line palliative chemotherapy for older adults.

The Korean version of the Nursing Delirium Screening Scale (Nu-DESC) was used for the screening of delirium in this study [32,33]. Five symptoms (disorientation, inappropriate behaviors, inappropriate communication, illusion/hallucinations, and psychomotor retardation) were evaluated by the Nu-DESC. Delirium is a common geriatric syndrome; abrupt neuropsychiatric disturbances that develop over hours, days, or weeks should be differentially diagnosed from preexisting cognitive impairments such as dementia [34,35]. It is reported that 26–44% of patients with advanced cancer show delirium, depending on the study consulted, and delirium is more prevalent in older patients. Various medical conditions can cause delirium, and up to 50% of cases are reversible [36,37]. Delirium can lead to misunderstandings and difficulties in treatment-related interventions or recognition when chemotherapy-associated toxicities occur. Delirium can also lead to changes in therapeutic strategies and may be associated with poor outcomes for patients with advanced cancer [38]. Therefore, the risk of delirium should be screened, and reversible factors of delirium also should be corrected in older adults with metastatic cancer.

Most of grade 3–5 adverse events tended to be more prevalent in the standard dose group than the UDR group (63.9% vs. 48.6%, *p* = 0.01) in our study. However oral mucositis was more frequently observed in the UDR group, which means oral mucositis may be associated with patient characteristics such as a poor performance status rather than drug exposure in this study. In the study by Gajra et al., chemotherapy toxicities of grades 3–5 were not associated with the dose of chemotherapy for older adults with cancer [5]. In terms of the toxicity assessments, we evaluated adverse events rather than treatment-related toxicity, because older adults have many comorbidities. In 2007, a report showed that a higher relative dose intensity (≥85%) was associated with higher neutropenic events in a study of patients ≥70 years old with major solid tumors, including lymphoma, accounting for 14% of all patients [39]. However, UDR should be performed very carefully in consideration of both its therapeutic efficacy and toxicities in the palliative setting, because UDR may reduce the therapeutic effects. It is important for the treating physician to estimate and identify the older adults who could suffer from severe chemotherapy toxicities before starting chemotherapy. Some studies reported tools for estimating chemotherapy toxicities in older adults with cancer [7,8,9,40]. However, these studies did not guide chemotherapy dosing in general. This treatment paradigm has been changing into precision medicine and immune oncology, and tools predicting adverse effects in to noncytotoxic therapy, such as hormonal agents, immunotherapy, and targeted therapy, should be also investigated. Grade 2 toxicities, which are more prevalent than grade 3 or 4 toxicities, should be considered and investigated for how these toxicities impact the variables of GA in older adults [41].

In our study, the frequencies of ER visits or hospitalization were higher in the standard dose group than the dose reduction group (52.9 % vs. 37.9 %, *p* = 0.01), and UDR was thought to be helpful with a better tolerance to chemotherapy in older adults with metastatic solid cancer. However, in the study by Gajra et al., more frequent hospitalizations were observed in the group of dose reductions [5]. These findings may be because the previous study included more frail patients who received different lines of chemotherapy compared with our study, which included only newly diagnosed patients. We found that the rate of study completion was higher in the UDR group than the standard dose group (29.4% vs. 16.8%, *p* = 0.02), and the relative dose intensity of chemotherapy, which was higher for the first 12 weeks in the standard group, was maintained higher in patients with UDR. These finding suggest that a “lower, longer” strategy of UDR seemed to help improve the older adults’ tolerance to chemotherapy compared with the standard dose group throughout the study. A standard dose could be considered at the second cycle for patients in the UDR group who presented with no toxicity during the inter-cycle period. However, the relative dose intensities for the following cycles of chemotherapy were not increased (Figure 2). The limitation of this study is that it was a retrospective study; in addition, the tumor type and chemotherapy regimen were heterogeneous, and this study dealt with only grade 3 or more adverse events. Furthermore, medical practice is in the era of noncytotoxic treatment, such as immunotherapy, hormonal therapy, and targeted agents, and grade 2 toxicities also should be considered. Therefore, a prospective disease-specific randomized study that expands the assessment of the adverse events to grade 2 is required in the future.

The establishment of tumor-specific and treatment regimen-specific clinical factors estimating the UDR of chemotherapy may be helpful for therapeutic planning for older adults with metastatic cancer. Our study revealed the prevalence of UDR of the first cycle of first-line palliative chemotherapy for older adults in clinical practice and the clinical characteristics of the patients who received UDR in multiple institutions of South Korea as the first study for older Asian adults with cancer. A prospective randomized trial to evaluate the benefits and drawbacks of UDR and the impact of UDR on adverse events and the efficacy of the subsequent chemotherapy for older adults with metastatic cancer should be performed.

## 4. Materials and Methods

### 4.1. Patients and Chemotherapy

A secondary analysis of data was performed in a multicenter prospective observational study for predicting chemotherapy toxicity from the geriatric assessment of older adults with cancer undergoing chemotherapy (KCSG PC13-09, WHO ICTRP number: KCT0001071) [3]. Among the 301 patients who were enrolled in 17 hospitals, a total of 296 patients were analyzed; this study excluded 5 patients, including 2 patients who withdrew consent and 3 patients who were not sure whether UDR was implemented. Eligible patients were 70 years or older, had a diagnosis of solid cancer, and were candidates for first-line palliative chemotherapy. Patients were excluded who had hematologic malignancy and received monotherapy with an oral chemotherapeutic drug, biological or targeted therapy alone, immunotherapy, or concurrent chemoradiotherapy. After written informed consent, enrolled patients received a GA before first-line palliative chemotherapy. The chemotherapy regimen was chosen at the oncologist’s discretion. The standard dose of chemotherapy was followed according to the recommendation of the National Comprehensive Cancer Network (NCCN) guidelines. Dose and schedule of the first-line chemotherapy was decided in clinical practice before the report of the results of the geriatric assessment. Patients were then categorized into UDR or standard dose groups. We defined UDR as any dose reduction during the first cycle of first-line chemotherapy from the recommended dose, which was less than the dose recommended for the reference regimens based on the NCCN guidelines. All patients who received the recommended doses of chemotherapy without dose reduction were considered in the group of standard dose chemotherapy. Two medical oncologists (IG Hwang and MS Kwon) reviewed each regimen and the recommended dosing to determine whether dose reduction occurred during the first cycle and to quantify the percent of dose reduction. When patients were treated with combination chemotherapy, they were defined as part of the UDR group if they received a reduced dose of any of their anticancer agents. The amount of UDR per patient was calculated by dividing the sum of the percent of the dose reduction in each chemotherapeutic agent by the number of all the chemotherapeutic agents used for the individual. For combination chemotherapy, the dose reduction was calculated as a mean of the percentage reduction for each agent. For example, for a FOLFOX regimen of 5-fluorouracil bolus, 5-fluorouracil continuous infusion, and oxaliplatin, if oxaliplatin was reduced by 25%, the 5-fluorouracil bolus by 20%, and 5-fluorouracil continuous infusion 0%, then the mean dose reduction was calculated as 15%. The calculated percent dose reduction was retrospectively confirmed by two medical oncologists.

### 4.2. Measurements

We evaluated the following in the patients ≥ 70 years receiving first-line palliative chemotherapy for solid tumors: (1) the prevalence of UDR; (2) differences in the clinical variables, including age; sex; performance status; body mass index (BMI); tumor type; geriatric assessment variables (comorbidities, activities of daily living (ADL), instrumental activities of daily living (IADL), cognitive functions, depression, delirium, the presence of a caregiver, and nutritional status); and the Korean Cancer Study Group Geriatric Score (KG)-7 between the two groups (UDR and standard dose patient groups); and (3) the relationship between adverse events and compliance (including emergency room (ER) visits or hospitalization, a delay of chemotherapy, and completion of the expected cycles of chemotherapy), according to the UDR. Adverse events were assessed using the National Cancer Institute Common Terminology Criteria for Adverse Events version 4.0 in each cycle of chemotherapy.

### 4.3. Statistical Analysis

Patient characteristics were analyzed by descriptive analyses for the mean, median, standard deviation (SD), range for continuous variables, and frequency for categorical variables. Statistical differences in the baseline characteristics between groups were evaluated by Fisher’s exact test or chi-square for the trends of the categorical variables and Student’s *t*-test or Wilcoxon rank sum test for the continuous variables. Before the *t*-test, a Kolmogorov–Smirnov test for normality was performed. Associations between the UDR and other clinical variables were also evaluated by Fisher’s exact test or chi-square for the trends. We performed bivariate logistic regression models with the considered predictors of UDR, which showed an association with a significance level of *p* < 0.1. Two-sided tests with a significance level of *p* < 0.05 were used. All statistical analyses were conducted using SPSS version 23 (IBM Corp., Armonk, NY, USA).

## 5. Conclusions

More than half of the older adults of aged ≥ 70 with metastatic solid cancers received UDR during the first cycle of the first-line palliative chemotherapy in clinical practice. Weighing the potential benefits and adverse events from cytotoxic chemotherapy is important in the care and management of older adults with cancer. In this retrospective study, poor PS and living without a spouse were independently associated with UDR, and patients with UDR were more tolerant throughout the first-line chemotherapy. A prospective randomized trial is required to confirm whether UDR is a beneficial therapeutic strategy for older adults with metastatic cancer and develop predictive factors to select adequate candidates who could benefit from UDR through the development of reliable predictive factors.

## Figures and Tables

**Table 1:** Patient characteristics.

Characteristic	Standard Dose (*N* = 119)	Upfront Dose Reduction (*N* = 177)	Total (*N* = 296)	*p*-Value
Age				0.15 ^※^
Mean (range)	74.8 (70–87)	75.5 (70–93)	75.0 (70–93)	
SD	3.7	4.1	4.00	
Sex				0.10 *
Male	89 (74.8%)	116 (65.5%)	205 (69.0%)	
Female	30 (25.2%)	61 (34.5%)	91 (30.7%)	
BMI				0.21 ^†^
Mean	22.8	22.3	22.5	
Range	15.7–31.2	13.9–29.6	13.9–31.2	
SD	3.2	3.0	3.1	
ECOG-PS				<0.01 *
0~1	106 (89.1%)	134 (75.7%)	240 (81.1%)	
2~4	13 (10.9%)	43 (24.3%)	56 (18.9%)	
Hypertension				0.23 *
Yes	62 (52.1%)	105 (59.3%)	167 (56.4%)	
No	57 (47.9%)	72 (40.7%)	129 (43.6%)	
Diabetes melitus				0.25 *
Yes	31 (26.1%)	58 (32.8%)	89 (30.1%)	
No	88 (73.9%)	119 (67.2%)	207 (69.9%)	
Cancer type				0.19 *
Colorectal	22 (18.5%)	62 (35.0%)	84 (28.4%)	
Lung	32 (26.9%)	42 (23.7%)	74 (25.0%)	
Biliary	17 (14.3%)	16 (9.0%)	33 (11.1%)	
Stomach	16 (13.4%)	16 (9.0%)	32 (10.8%)	
Pancreas	12 (10.1%)	14 (7.9%)	26 (8.8%)	
GU	5 (4.2%)	11 (6.2%)	16 (5.4%)	
Head and Neck	4 (3.4%)	4 (2.3%)	8 (2.7%)	
Breast	2 (1.7%)	1 (0.6%)	3 (1.0%)	
GYN	2 (1.7%)	1 (0.6%)	3 (1.0%)	
Other	7 (5.9%)	10 (5.6%)	17 (5.7%)	
Hemoglobin, g/dL				1.00 *
≥10 (female), ≥11 (male)	90 (75.6%)	134 (75.7%)	224 (75.7%)	
<10 (female), <11 (male)	29 (24.4%)	43 (24.3%)	72 (24.3%)	
WBC, ×10^3^/μL Median (range)	7.0 (2.9–27.8)	7.44 (3.43–20.5)	7.3 (2.9–27.7)	0.203 ^‡^
Platelet, ×10^3^/μL Median (range)	244 (79–493)	257 (103–636)	252 (79–636)	0.195 ^‡^
AST, IU/mL Median (range)	23 (11–96)	22 (7–239)	22 (7–239)	0.840 ^‡^
Albumin, g/dL Median (range)	3.7 (2.3–4.5)	3.7 (2.2–4.6)	3.7 (2.3–4.6)	0.369 ^‡^
Estimated GFR				0.114 *
≥60 mL/min/1.73 m^2^	99 (83.2%)	133 (75.1%)	232 (78.4%)	
<60 mL/min/1.73 m^2^	20 (16.8%)	44 (24.9%)	64 (21.6%)	
C-reactive protein, mg/L Median (range)	1.29 (0.01–111.84)	1.05 (0.01–191.58)	1.14 (0.01–191.58)	0.376 ^‡^

Abbreviations: BMI, Body mass Index; SD, Standard deviation; ECOG-PS, Eastern Cooperative Oncology Group Performance Status; GU, Genitourinary cancer; GYN, Gynecologic cancer; WBC, white blood cell; AST, aspartate aminotransferase; and GFR, glomerular filtration rate. The estimated GFR was calculated with the Berlin Initiative Study equation using the serum creatinine (BIS-1). ^※^ *t*-test. * Fisher’s exact test. ^†^ Mann–Whitney U test. ^‡^ Kruskal-Wallis test.

**Table 2:** Association between upfront dose reduction and variables of geriatric assessment.

Variables	Standard Dose (*N* = 119)	Upfront Dose Reduction (*N* = 177)	Total (*N* = 296)	*p*-Value
Comorbidity (Charlson risk index)				0.30 ^†^
Low (0 points)	61 (51.3%)	93 (52.5%)	154 (51.7%)	
Medium (1 to 2 points)	42 (35.3%)	70 (39.5%)	112 (38.1%)	
High (3 to 4 points)	14 (11.8%)	14 (7.9%)	28 (9.5%)	
Very high (≥5 points)	2 (1.7%)	0 (0.0%)	2 (0.7%)	
Activities of daily living				<0.01 *
Independent	96 (80.7%)	115 (65.0%)	211 (71.3%)	
Dependent	23 (19.3%)	62 (35.0%)	85 (28.7%)	
Instrumental activities of daily living (KIADL)				0.55 *
Independent	71 (59.7%)	99 (55.9%)	170 (57.4%)	
Dependent	48 (40.3%)	78 (44.1%)	126 (42.9%)	
Cognitive function (MMSE-KC)				0.77 ^†^
Intact (25–30)	48 (40.3%)	83 (46.9%)	131 (44.3%)	
Mild impairment (17–24)	62 (52.1%)	73 (41.2%)	135 (45.6%)	
Severe impairment (≤16)	9 (7.6%)	21 (11.9%)	30 (10.1%)	
Depression (SGDS)				0.39 ^†^
Intact (<5)	69 (58.0%)	95 (53.7%)	164 (55.4%)	
Mild depression (5–9)	38 (31.9%)	52 (29.4%)	90 (30.4%)	
Severe depression (≥10)	11 (9.2%)	29 (16.4%)	40 (13.5%)	
Not available	1 (0.8)	1 (0.6%)	2 (0.7%)	
Delirium risk screen (Korean Nu-DESC)				<0.01 *
No risk	117 (98.3%)	160 (90.4%)	277 (93.6%)	
With Risk	2 (1.7%)	17 (9.6%)	19 (6.4%)	
Nutritional status (MNA)				0.83 ^†^
Normal (≥24)	28 (23.5%)	41 (23.2%)	69 (23.3%)	
Risk of malnutrition (17–23)	72 (60.5%)	96 (54.2%)	168 (56.8%)	
Malnutrition (<17)	18 (15.1%)	40 (22.6%)	58 (19.6%)	
Not available	1 (0.8%)	0 (0.0%)	1 (0.3%)	
Living alone				0.13
Yes	12 (10.1%)	30 (16.9%)	42 (14.2%)	
No	107 (89.9%)	147 (83.1%)	254 (85.8%)	
Living with a spouse				<0.01 *
Yes	96 (80.7%)	118 (66.7%)	214 (72.3%)	
No	23 (19.3%)	59 (33.3%)	82 (27.7%)	
Male				0.02 *
Yes	82 (92.1%)	93 (80.2%)	175 (85.4%)	
No	7 (7.9%)	23 (19.8%)	30 (14.6%)	
Female				0.66 *
Yes	14 (46.7%)	25 (41.0%)	39 (42.9%)	
No	16 (53.3%)	36 (59.0%)	52 (57.1%)	

* Fisher’s exact test. ^†^ chi-square test. Korean Nu-DESC, Korean version of the Nursing Delirium Screening Scale.

**Table 3:** Multivariate analysis of the factors associated with upfront dose reduction.

	Univariate Analysis			Multivariate Analysis		
Variables	OR	(95% CI)	*p*-Value	OR	(95% CI)	*p*-Value
Poor ECOG-PS (2~4 vs 0~1)	2.62	(1.34–5.12)	0.01	2.33	(1.17–4.64)	0.02
Dependent in activity of daily living	2.25	(1.30–3.90)	<0.01			
Not living with a spouse	2.09	(1.02–3.62)	0.01	1.89	(1.07–3.33)	0.03
High delirium risk	6.22	(1.41–27.43)	0.02	4.44	(0.98–20.21)	0.05

Abbreviations: OR, Odds ratio; CI, Confidence interval; and ECOG-PS, Eastern Cooperative Oncology Group Performance.

**Table 4:** Grade 3–5 adverse events.

Variables	Standard Dose (*N* = 119)	Upfront Dose Reduction (*N* = 177)	Total (*N* = 296)	*p*-Value
Any Grade 3	70 (58.8%)	80 (45.2%)	150 (50.7%)	
Any Grade 4	34 (28.6%)	25 (14.1%)	57 (19.3%)	
Any Grade 5	7 (5.9%)	8 (4.5%)	13 (4.4%)	<0.001 ^†^
Any Grade 3–5	76 (63.9%)	86 (48.6%)	162 (54.7%)	0.01 *
**Hematologic Adverse Events (Grade 3–5)**				
Neutropenia	44 (37.0%)	37 (20.9%)	83 (27.4%)	<0.001 *
Febrile Neutropenia	6 (5.0%)	7 (4.0%)	13 (4.4%)	0.77 *
Leukopenia	10 (8.4%)	11 (6.2%)	21 (7.1%)	0.50 *
Anemia	18 (15.1%)	15 (8.5%)	33 (11.1%)	0.04 *
Thrombocytopenia	14 (11.8%)	9 (5.1%)	23 (7.8%)	0.04 *
**Nonhematologic Adverse Events (Grade 3–5)**				
Fatigue	6 (5.0%)	15 (8.5%)	21 (7.1%)	0.13 *
Generalized muscle weakness	7 (5.9%)	1 (0.6%)	7 (2.4%)	0.01 *
Thromboembolism	3 (2.5%)	4 (2.3%)	7 (2.4%)	1.00 *
Oral Mucositis	0 (0)	7 (4.0%)	7 (2.4%)	0.04 *
Peripheral sensory neuropathy	0 (0)	2 (1.1%)	2 (2.4%)	0.52 *
Anorexia	12 (10.1%)	8 (4.5%)	20 (6.8%)	0.09
Nausea	4 (3.8%)	10 (5.3%)	14 (6.7%)	0.41 *
Vomiting	3 (2.5%)	2 (1.1%)	5 (1.7%)	0.39 *
Diarrhea	4 (3.4%)	6 (3.4%)	10 (3.4%)	1.00 *
Infection	11 (9.2%)	9 (5.1%)	10 (6.8%)	0.23 *
Sepsis	3 (2.5%)	3 (1.7%)	6 (2.0%)	0.68 *
Arrhythmia	2 (1.7%)	3 (1.7%)	5 (1.7%)	1.00 *

* Fisher’s exact test. ^†^ chi-square test.

**Table 5:** The effect of a cisplatin-containing regimen on the hematologic adverse events (Grade 3–5).

Variables	Standard Dose (*N* = 119)	Upfront Dose Reduction (*N* = 177)	Total (*N* = 296)	*p*-Value
Neutropenia	44 (37.0%)	37 (20.9%)	83 (27.4%)	
Cisplatin (−)	28/99 (28.3%)	30/133 (22.6%)	58/232 (27.2%)	0.319
Cisplatin (+)	16/20 (80.0%)	7/44 (15.9%)	23/64 (28.1%)	0.016
Anemia	18 (15.1%)	15 (8.5%)	33 (11.1%)	
Cisplatin (−)	7/99 (7.1%)	12/133 (9.0%)	26/232 (11.2%)	0.917
Cisplatin (+)	11/20 (55.0%)	3/44 (6.8%)	7/64 (10.9%)	0.012
Thrombocytopenia	14 (11.8%)	9 (5.1%)	23 (7.8%)	
Cisplatin (−)	4/99 (4.0%)	6/133 (4.5%)	19/232 (8.2%)	0.774
Cisplatin (+)	10/20 (50.0%)	3/44 (6.8%)	4/64 (6.3%)	0.022

**Table 6:** Tolerance of and compliance with first-line palliative chemotherapy.

Variables	Standard Dose (*N* = 119)	Upfront Dose Reduction (*N* = 177)	Total (*N* = 296)	*p*-Value
ER visit or hospitalization				
Yes	63 (52.9%)	67 (37.9%)	130 (43.9%)	
No	56 (47.1%)	110 (62.1%)	166 (56.1%)	0.01 *
Number of patients with delayed chemotherapy				
Yes	61 (51.3%)	88 (49.7%)	149 (50.3%)	
No	58 (48.7%)	89 (50.3%)	147 (49.7%)	0.80 *
Number of cycles with delayed chemotherapy				
Median	3	1	1	0.39 ^†^
Range	1–15	1–6	0–15	
Delayed dates of chemotherapy (day)				
Median	14.5	22	1	0.65 ^†^
Range	1–67	2–117	1–117	

Abbreviations: ER, Emergency room. * Fisher’s exact test. ^†^ Kruskal-Wallis test.

**Table 7:** The completion of first-line palliative chemotherapy as planned.

Variables	Standard Dose (*N* = 119)	Upfront Dose Reduction (*N* = 177)	Total (*N* = 296)	*p*-Value *
Study Completion	20 (16.8%)	52 (29.4%)	72 (24.3%)	0.02
Not completed	99 (83.2%)	125 (70.6%)	224 (75.7%)	
Death	69 (58.0%)	97 (54.8%)	166 (56.1%)	0.63
Follow-up loss	12 (10.1%)	12 (6.8%)	24 (8.1%)	0.18
Patient decision	8 (6.7%)	12 (6.8%)	20 (6.8%)	1.00
Transfer	10 (8.4%)	4 (2.3%)	14 (4.7%)	0.02

* Fisher’s exact test.

## Data Availability

The data presented in this study are available on request from the corresponding author. The data are not publicly available due to a privacy issue from the patients.
